# Low FODMAP Diet: Evidence, Doubts, and Hopes

**DOI:** 10.3390/nu12010148

**Published:** 2020-01-04

**Authors:** Massimo Bellini, Sara Tonarelli, Attila G. Nagy, Andrea Pancetti, Francesco Costa, Angelo Ricchiuti, Nicola de Bortoli, Marta Mosca, Santino Marchi, Alessandra Rossi

**Affiliations:** 1 Gastrointestinal Unit–Department of Translational Sciences and New Technologies in Medicine and Surgery, University of Pisa, 56124 Pisa, Italy; satonarelli@gmail.com (S.T.); n.attilagabor@gmail.com (A.G.N.); pancio10@alice.it (A.P.); fcosta@med.unipi.it (F.C.); a.ricchiuti@int.med.unipi.it (A.R.); nicola.debortoli@unipi.it (N.d.B.); santino.marchi@unipi.it (S.M.); 2 Clinical and Experimental Medicine–Rheumatology Unit, University of Pisa, 56100 Pisa, Italy; marta.mosca@med.unipi.it (M.M.); alessandra.rossi@unipi.it (A.R.)

**Keywords:** irritable bowel syndrome, low FODMAP diet, nutrition, gut microbiota

## Abstract

Food is often considered to be a precipitating factor of irritable bowel syndrome (IBS) symptoms. In recent years, there has been a growing interest in FODMAPs (Fermentable Oligo-, Di-, Mono-saccharides, And Polyols), which can be found in many common foods. A low FODMAP diet (LFD) is increasingly suggested for IBS treatment. However, long-term, large, randomized controlled studies are still lacking, and certainties and doubts regarding LFDs have grown, often in a disorderly and confused manner. Some potential LFD limitations and concerns have been raised, including nutritional adequacy, cost, and difficulty in teaching the diet and maintaining it. Most of these limitations can be solved with the involvement of a skilled nutritionist, who can clearly explain the different phases of the LFD and ensure nutritional adequacy and compliance. Further studies should focus on new methods of teaching and learning the LFD and on predictors of response. Moreover, particular interest should be focused on the possible use of LFD in gastrointestinal diseases other than functional disorders and, possibly, also in non-gastrointestinal diseases. The aim of the present review was to clarify the effective and appropriate indications and limitations of an LFD and to discuss its possible future uses.

## 1. Introduction

In approximately 60% of cases, patients affected with irritable bowel syndrome (IBS) have reported eating as a precipitant of their symptoms, which deeply impacts their quality of life [1,2,3]. Furthermore, it is well-known that patients with a higher degree of self-reported food intolerance show more severe IBS symptoms [4].

In 1959, hypolactasia was the first reported disorder caused by impaired saccharide absorption [5]. Since then, many other saccharides have been documented as causing gastrointestinal (GI) symptoms. In recent years, there has been a growing interest in a wide class of foods, grouped under the acronym FODMAPs, which stands for Fermentable Oligo-, Di-, Mono-saccharides, And Polyols. FODMAPs are a large class of small nondigestible carbohydrates, containing only 1–10 sugars which are poorly absorbed in the small bowel. FODMAPs can be found in a range of very common and different foods such as fruits, vegetables, legumes and cereals, honey, milk and dairy products, and sweeteners [6].

All FODMAPs are potential triggers, but, fortunately, not all FODMAPs exacerbate abdominal symptoms in the same IBS patient.

The total daily intake of FODMAPs in a habitual diet ranges from 15 grams to 30 grams per day. For a long time, the restriction of individual FODMAPs has been used with varying success in the management of IBS (e.g., restriction of lactose and/or gluten and/or some vegetables and/or some cereals) [7,8,9].

However, an LFD is not only a gluten- and lactose-free diet. It is a more of a global restriction, which has a greater and more consistent effect than a more limited diet [10]. Therefore, for example, a single pear may not cause an IBS flare up, but consuming some slices of bread, half an onion, a pear, and a glass of milk during the same meal may cause the onset of IBS symptoms.

The low-FODMAP approach is not simply an “avoidance diet”. It is also a diagnostic tool to test the patients’ tolerance to some foods, enabling the patients to eliminate them from their diet and to make significant changes to their lifestyle.

**Figure 1:**
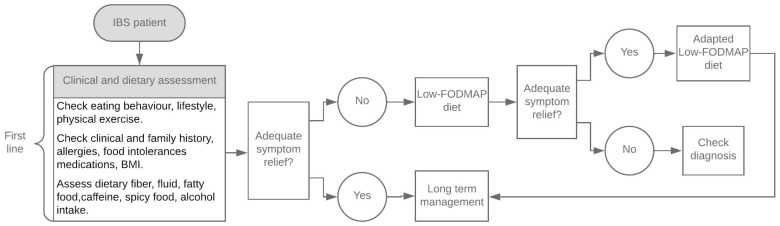
Dietary management algorithm in irritable bowel syndrome (IBS), modified from McKenzie YA, 2016 [12].

An LFD involves a global restriction of FODMAP intake for four to eight weeks, followed by their gradual reintroduction according to individual tolerance. This enables personalization of the diet and its implementation in the long-term (Figure 1). To ensure nutritional adequacy in the long-term, it is essential that the reintroduction phase is closely monitored by a qualified and skilled nutritionist [11].

The mechanisms of action of FODMAPs are mainly linked to their osmotic activity, forcing water into the GI tract [13]. Moreover, after entering the colon, they represent a food that is easy to use by the intestinal microbiota, which ferment them and thus increase gas production. This, in turn, worsens luminal distension. Furthermore, it has been proven that an LFD also acts by activating Meissner’s plexus, modulating the neuroenteric sensory transmission, which stimulates intestinal secretion and motility and accelerates transit time [14].

FODMAP action on the gut can be summarized as follows:

Increasing small bowel water content: One randomized single-blind cross-over trial showed that a high-FODMAP Diet (HFD; 112 g/day) increased the intestinal water content in patients with ileostomy, even if the increase was found to be lower than the maximal colonic water quantity tolerated by healthy individuals [15,16].

Increasing production of gas: FODMAP fermentation leads to a larger production of gases such as hydrogen, methane, and carbon dioxide, as detected in a trial using magnetic resonance imaging by Murray et al. [17]. Nevertheless, a recent crossover study suggested that an LFD could lead to a reduced production of gas through modifications of the microbiota, with a relative increase in the hydrogen-consuming bacterial genus *Adlercreutzia* [18].

Excessive production of short-chain fatty acids (SCFAs): SCFAs (propionate, butyrate, and acetate) are products of bacterial dietary fiber metabolism and display many beneficial effects. Butyrate, in particular, is the most important energy source for the colonic epithelium and has a range of effects relevant to the maintenance of gut epithelial health [19]. Moreover, propionate and acetate may also have systemic immunomodulatory and epigenetic effects. However, butyrate can increase visceral sensitivity, as shown by Vanhoutvin in healthy volunteers [20]. SCFAs can be toxic to the epithelium if present in high concentrations and, by stimulating the release of 5-hydroxytryptamine (5HT) from the intestinal mucosa, they favor the onset of high-amplitude propagated colonic contractions, thus accelerating intestinal transit [21].

These phenomena, taken as a whole within the context of the visceral hypersensitivity typical of IBS patients, may provoke abdominal pain, bloating, flatulence, and alterations in bowel habits.

Despite the high number of papers published in recent years, certainties and doubts regarding LFDs have often emerged in a disorderly and confused manner. The aim of the present review was to contribute to clarifying the effective and appropriate indications and limitations of a LFD and to discuss its possible future uses.

## 2. Evidence 

An LFD is not a panacea for all patients affected by GI disturbances, but there are now several studies proving the efficacy of LFDs as a treatment for IBS.

A meta-analysis by Marsh et al., analyzed six randomized controlled trials (RCTs) and 16 non-RCTs supporting the efficacy of the LFD as a treatment for functional gastrointestinal symptoms [22]. There was a significant decrease in the score of the Irritable Bowel Syndrome Symptom Severity Scale (IBS-SSS) and an improvement in abdominal pain, bloating, and Irritable Bowel Syndrome Quality of Life (IBS-QOL). In the non-RCTs diarrhea, nausea and constipation were found to improve. Another meta-analysis by Schumann et al., included nine RCTs with a total of 596 patients. They found that an LFD, in comparison to other diets, i.e., habitual diet, diet high in FODMAPs (HFD), sham diet, and usual dietary recommendations for IBS, was effective and safe in the short-term [23].

A recent systematic review provided evidence that a LFD is effective in reducing IBS symptoms [24]. Although the evidence is of very low quality, an LFD had the greatest efficacy among dietary interventions suggested for treating IBS symptoms. The authors justified these results because of the different types of comparator groups used in the various trials and the relatively low number of patients reporting a global symptom improvement (189 patients, whereas the GRADE system would require at least 300 patients) [25]. The authors also underlined that the problems could be solved if further trials could be carried out using similar comparator groups in order to provide more data. Unfortunately, there is a problem of economic resources because it is quite difficult to find subjects interested in financing such studies.

Only a few studies dealing with LFDs have been based on randomized placebo-controlled double-blind trials. In Table 1, we provide an indication of the quality of the evidence based on the study design [25]. Moreover, most trials aimed at demonstrating the efficacy of LFD in the short-term, and long-term studies are still lacking (Table 1).

An LFD is usually compared with other kinds of diets (i.e., habitual diet, standard IBS diet, sham diet, HFD) or with other treatments. The majority of clinical trials suggest that an LFD provides significant symptom relief in comparison with other diets, even though the parameters used for evaluating the improvement were often different among the various studies. The IBS-SSS is the most widely used measurement assessing symptom severity in the aforementioned trials [45]. Alternative measures to evaluate LFD efficacy are the visual analogue scale (VAS) and numerical rating scale of different symptoms, the degree and the adequacy of symptom relief, and the GI Symptom Rating Scale [46,47]. As reported above, these evaluation differences are a potential source of bias that deserves to be further discussed.

### 2.1. LFD Compared to Other Diets

A single-blind cross-over trial by Ong et al., which lasted for only two days and supplied all the food needed for the purpose of the study to the patients, compared an LFD with a HFD [26]. It clearly showed a difference between the two groups in favor of the LFD, both in terms of gastrointestinal symptoms and gas production, calculated with the hydrogen and methane breath test.

A randomized controlled trial by Staudacher et al. compared an LFD with a habitual diet for four weeks in a group of 41 IBS patients (19 in the LFD intervention and 22 in the control group) [27]. Symptom control was assessed with a global question regarding satisfaction with symptoms. Furthermore, a more precise assessment was obtained using patient diaries based on the GI Symptom Rating Scale. Among the LFD group patients, 68% had adequate symptom control compared to 23% in the control group (*p* = 0.005). In the intention-to-treat population, more patients in the intervention group experienced a reduction in scores for bloating (*p* = 0.007), borborygmi (*p* = 0.04), urgency (*p* = 0.047), and overall symptoms (*p* = 0.006) compared with the control group.

Pedersen et al. compared an LFD with a typical Danish diet and with the administration of *Lactobacillus rhamnosus GG* (LGG) in a group of 123 IBS patients over a period of six weeks [28]. They reported a significant reduction in IBS-SSS both in the LFD and the LGG arms.

A placebo-controlled randomized trial was carried out in 30 IBS patients for 21 days, comparing an LFD with a typical Australian diet [29]. All of the food was provided to the patients. At the end of the trial, the VAS in the LFD group was of 22.8 mm (*p* < 0.001), as opposed to 44.9 mm in the typical Australian diet group. Approximately 70% among the LFD patients reported a reduction greater than 10 mm on the VAS of their IBS symptoms (overall gastrointestinal symptoms, bloating, pain, passing of wind, and dissatisfaction with stool consistency).

A Swedish randomized study showed that only 50% of 84 IBS patients responded to an LFD after a four week treatment in terms of IBS-SSS and frequency of bowel movements, with similar responses in the LFD and a traditional IBS diet, which, however, also reduced the intake of some FODMAPs [30].

Chumpitazi et al. carried out a blind all-feed trial on a pediatric group, comparing an LFD with a Typical American Childhood Diet (TACD) high in fructose [31]. This trial showed fewer episodes of abdominal pain and minor severity during the LFD compared to the TACD. Interestingly, they found microbiota differences in stool samples between responders and non-responders to the LFD. This could shed light on an important and largely unexplored aspect of LFD, such as their potential use as predictors of response.

One of the diets recognized as a “standard” diet for treating IBS is the UK National Institute for Health and Clinical Excellence (NICE) diet [48]. This is considered to be a first line dietary therapy for IBS, and it has never been evaluated in placebo-controlled trials. It is based on some suggestions regarding eating behaviors, such as having regular meals, reducing the intake of alcohol, and drinking at least eight cups of fluids per day, etc. There are also some food restrictions that are included in the LFD approach, such as the reduction of ‘resistant starch’, sorbitol, and whole wheat products. A non-randomized trial compared the NICE diet for IBS with an LFD in 82 patients over nine months [37]. There was greater overall satisfaction and less bloating and abdominal pain in 86% of patients who carried out the LFD, compared to 49% of those who followed the NICE diet (*p* < 0.001).

A randomized controlled trial involving 84 diarrhea-predominant IBS patients (IBS-D) in the US showed a significantly greater improvement in abdominal pain and bloating in the group of patients who followed an LFD for four weeks [32]. This compared well with patients who had followed a modified NICE diet (not excluding high-FODMAP foods).

In a randomized double-blind controlled cross-over study, 87 IBS participants were supplied with regular rye bread (high in FODMAPs) or with a low-FODMAP bread [33]. Apart from this, they had to follow their regular diet. After four weeks, many IBS symptoms were less severe (flatulence *p* = 0.04, abdominal pain *p* = 0.049, cramps *p* = 0.01, stomach rumbling *p* = 0.001) in the low-FODMAP rye bread arm, but there were no differences in IBS-SSS or IBS-QOL. This could be explained by the fact that the study tested only the rye bread, and the effects of other molecules were not investigated at all within the context of a complete LFD.

A Canadian study compared a dietitian-led LFD with am HFD [18]. The study, lasting three weeks, showed that 72% of LFD patients improved their IBS-SSS and decreased the production of histamine compared to 21% HFD patients. This was calculated by metabolic profiling of urine, which is an important signaling molecule for the genesis of IBS symptoms [21].

In order to enhance blindness, a randomized placebo-controlled trial was performed by Staudacher et al. using a placebo (sham) diet similar in amount of food restriction and in difficulty of continuation to an LFD [34]. The trial took four weeks and 104 IBS patients were enrolled. The incidence and severity of 15 GI symptoms and of overall symptoms were measured. A significantly higher proportion of patients on the LFD had adequate symptom relief (61%) compared to those on the sham diet (39%) (*p* = 0.042). Total mean IBS-SSS was significantly lower in the LFD group (173 ± 95) than in the control group (224 ± 89) (*p* = 0.001). Although the endpoint of “adequate symptom relief” in the intention-to-treat analysis was not significant, the study suggests that the LFD reduced symptoms compared with the placebo diet when evaluated with more precise methods such as the IBS-SSS questionnaire.

Hustoft et al. carried out a double-blind cross-over trial involving 20 patients with diarrheic IBS (IBS-D) or mixed IBS (IBS-M) [35]. After a three-week LFD, randomized patients received either a supplement of fructooligosaccharides or maltodextrin (placebo) for 10 days. The symptoms improved after the three-week LFD, and a greater number of patients continued to have symptom control with the placebo. This compared well with the patients supplemented with fructooligosaccharides, which was assessed with the IBS-SSS questionnaire, additional GI complaints, and a comorbidities questionnaire.

### 2.2. LFD Compared to Other Therapies

Only a few studies have compared an LFD with other therapies currently used for IBS treatment.

Gut-directed hypnotherapy has shown evidence of efficacy in a number of clinical trials, and it is now considered a valuable option for treating IBS patients [36,49]. Hypnotherapy could help in improving IBS symptoms by providing better coping strategies for psychological problems. Indeed, it is recommended for IBS by NICE in patients not responding to dietary and pharmacological treatments. Probably these “refractory” patients have a more complex form of IBS and a greater psychological involvement [50].

A randomized trial proved that the efficacy of an LFD is similar to that of hypnotherapy, with a positive response in GI (pain, bloating, wind, stool consistency, and nausea) and psychological symptoms (anxiety and depression) after six weeks in 71% of patients [38]. The efficacy was also maintained after six months with no statistical difference among the groups treated with hypnotherapy alone, among the diet group and a combination of the two (74%, 82%, and 54% respectively). This proved the long-term effectiveness of both the LFD and hypnotherapy. Surprisingly, the combination of the LFD and hypnotherapy resulted in a lower percentage of long-term efficacy. The authors hypothesized that this effect could be due to a possible placebo effect not investigated by their study, or to a “ceiling effect” of the symptom evaluation method (VAS). Alternatively, it could perhaps be due to a possible negative interplay between the two therapeutic choices.

It is not clear why yoga might be active regarding IBS symptoms. A positive action on the brain gut axis and on stress could be possible, as well as an improvement in the quality of sleep and of life. A recent systematic review, taking into account about 100 papers, identified only six articles regarding yoga, with a total of 273 patients [51]. There was a practical impossibility of carrying out a metanalysis because of the different enrollment criteria and endpoints, the different combination of yoga exercises, different comparators, difficulty in having a control group, difference in follow-up length, etc. There was evidence for a positive effect on IBS symptoms and anxiety, even though two of the studies examined did not find any difference in comparison with conventional treatments. The authors concluded that yoga could be considered a safe, but only adjunctive, treatment for IBS patients.

A single-blind RCT by Schumann et al. compared treatment with an LFD with yoga for 12 weeks in 59 IBS patients [39]. Both groups had a statistically significant reduction in the IBS-SSS (the total score and the subcategories: Duration of pain, bowel satisfaction, interference with life) after 12 weeks of active intervention. The improvement lasted also until the end of a 12-week follow-up.

### 2.3. LFD Beyond IBS

As recently reported by Colombel et al., an LFD may be offered to manage the functional GI symptoms in IBD [52]. This is because FODMAPs could be responsible for functional symptoms in patients with quiescent IBD, and some studies seem to confirm the possible efficacy of a LFD [53].

Prince et al. examined the impact of a six-week LFD on functional-like symptoms in 88 IBD patients in the absence of active inflammation [54]. Evaluation by means of the Gastrointestinal Symptom Rating Scale showed a relief of symptoms in 78% of patients. There was a particularly significant reduction in the severity of abdominal pain, bloating, flatulence, belching, incomplete evacuation, nausea, and heartburn, as well as an increase in the number of patients reporting normal stool consistency (55/88, 63%).

Gibson showed that an LFD was beneficial in about 50% of IBD patients with abdominal symptoms in the quiescent phase of the disease [55].

As shown by these studies, an LFD seems to be effective on symptoms of a functional nature and few data on the anti-inflammatory effect of LFDs are currently available.

LFDs, through their action on the composition of the intestinal microbiota and on the consequent production of bacterial metabolites such as SCFAs, are considered by some authors to be potentially harmful, especially on a fragile balance as in the case of IBD patients [53].

A recent meta-analysis concluded that an LFD is effective in reducing GI symptoms in patients with quiescent IBD. However, this does not seem to be due to a further improvement in intestinal inflammation, because no differences in fecal calprotectin were found between IBD patients on a LFD in comparison with those on a standard diet [56,57].

Bodini et al., in a recent randomized trial on 55 IBD patients, reported that a six-week LFD led to a decrease in calprotectin values and in disease activity as assessed by the Harvey-Bradshaw Index and partial Mayo Score [58]. It also led to a slight improvement in quality of life. However, according to the authors, this decrease in the value of fecal calprotectin should be addressed not as an intrinsic anti-inflammatory activity of the diet, but rather as a lack of potential worsening of the disease, suggesting efficacy and safety in this kind of patient.

Another field that should be further explored is nonceliac gluten sensitivity (NCGS). Biesiekierski et al., in a consecutive double-blind, randomized, placebo-controlled, cross-over re-challenge study, showed that NCGS patients treated with a LFD improved GI symptoms and reduced fatigue [59]. They did not find any evidence that gluten induced symptoms in NCGS patients when put on an LFD. They concluded that gluten could trigger symptoms only in the presence of a moderate amount of FODMAPs because gluten-containing cereals are high in fructans, which are FODMAPs, so their reduction leads to an improvement in symptoms.

An LFD could also be offered to patients affected with non-GI diseases. GI involvement in systemic sclerosis (SSc) is quite common, and patients often ask for dietary suggestions to improve their GI symptoms. To date, the evidence regarding a possible efficacy of dietary modifications has been scarce, but in small, non-randomized, uncontrolled open-label trials, improvements in gastrointestinal symptoms have been reported after using probiotics and a LFD [60,61]. Also, in fibromyalgia patients, Marum et al. showed that an LFD could be effective in reducing GI symptoms and fibromyalgia symptoms, such as somatic pain and muscle tension, and in improving the quality of sleep, anxiety, depression, and asthenia [62,63].

## 3. Doubts

Some potential limitations and concerns of LFDs have been raised and partially remain to be clarified [64]. They are amplified in patients who follow the diet without professional advice. Indeed, the presence of a skilled nutritionist is extremely important in:

- Explaining the nature and the aim of the diet;
- Ensuring nutritional adequacy and avoiding nutritional and caloric imbalance;
- Favoring patients’ compliance with frequent monitoring (e.g., phone calls, emails) and giving timely suggestions;
- Adapting the diet to the normal eating behavior and lifestyle (personal taste, ethnicity, etc.) of the patients.

Dietary intake may be analyzed on a qualitative basis via a dietary history taken by a dietitian. It may be also analyzed semi-quantitatively using tables of known FODMAP content [65]. In a recent paper, we applied the cutoff values to individual food (per serving) by means of the published FODMAP table content [7,8,9,66,67].

The skills of a dietitian in dietary assessment, knowledge of FODMAP food composition, and experience with the LFD approach are likely to impact positively on the success of the diet [68].

The real, or presumed, problems for the patient who wants to go on an LFD are listed below.

### 3.1. Complex and Difficult to Teach and to Learn 

An LFD is relatively complex, and patients benefit from being provided with an explanation of the mechanism of action. It is not an “easy” diet, and problems increase when patients follow LFDs without any professional advice [11]. Medical practitioners (and gastroenterologists) often have a limited knowledge of LFDs and they might be over-restrictive (e.g., advising the patient to avoid any kind of dairy product due to lactose malabsorption) or under-restrictive (e.g., advising a restricted intake of fructose alone). Moreover, they may have limited resources and insufficient time to teach a diet, resorting to diet sheets that might be out of date.

A recent study by Trott et al. showed how general practitioners in the UK, who are often the first contact of the IBS patient with the health system, do not have the knowledge to propose the LFD without the risk of encountering several nutritional risks, such as causing a micronutrient deficiency micronutrients or an unnecessary weight loss [69]. Educational groups could be timesaving for health professionals and could be useful for improving the patients’ knowledge of LFDs.

### 3.2. Difficult to Continue and Potentially Expensive

Patients’ adherence is crucial for the success of any kind of diet [40,65].

Gearry et al. suggested that adherence is positively associated with the improvement of symptoms and is related to the use of FODMAP-specific cookbooks, which provide variety in the diet and make it easier to follow [70]. It is important to find good recipes because some patients describe the diet as too bland. Moreover, the authors sustained that a higher education and working no more than 35 hours per week are factors which bring about a better understanding and adherence to the diet.

It is also reported that people with few economic resources may have difficulty in adherence, even for short periods of time [70]. Moreover, the problem of a lack of standardization of food labels could increase the difficulties met by patients [71]. This is because most countries do not report FODMAP food content.

However, only a few studies have investigated patients’ adherence to an LFD and its acceptability. Maargard et al. carried out a retrospective cross-sectional study involving 131 IBS and 49 inflammatory bowel disease (IBD) patients with coexisting IBS symptoms [41]. They found that, at a median follow-up of 16 months, about one-third of patients were adherent to the LFD, about 30% followed the diet for less than three months, about 50% were still on the diet at follow-up, and 54% used the LFD on and off. At follow-up, 84% undertook a modified LFD where some tolerated foods were reintroduced, while 16% of patients followed the usual LFD. The most common reasons for giving up the diet before the end of the LFD period were increased difficulty in continuing the diet, high cost, and bland taste.

O’Keeffe reported that also a less restrictive LFD after the reintroduction phase (adapted LFD: aLFD) was found to be more expensive than the patient’s habitual diet [42]. Furthermore, the patients met increasing difficulty in eating at friends’ houses, in restaurants, and while travelling. However, no significant difference was found regarding the food-related QOL between the aLFD and a habitual diet.

Indeed, the burden associated with following a complex diet could limit the impact of the diet’s positive influence or could indeed adversely impact on patients’ Health Related Quality of Life (HRQOL) [72]. A limited number of studies have directly measured HRQOL in response to an LFD with conflicting results. Some studies demonstrated improvement in disease-specific HRQOL after dietary advice [38,41,71], while others showed no effect [28].

Pedersen et al. studied 19 IBS patients and found significant differences in IBS-QOL, which improved significantly only in IBS-D patients when they were following the LFD [73].

Peters et al. did not find any significant difference in the improvement of the IBS-QOL of patients receiving gut directed hypnotherapy, LFD, or a combination of the two treatments, showing that the quality of life had significantly improved in all groups [38].

In its initial period, an LFD in can be difficult to follow and is a little more expensive. However, in the medium- and long-term, patients are able to deal with these issues.

### 3.3. Reduction of Natural Prebiotics and Impact on Gut Microbiota and Metabolome 

The restriction of FODMAPs can reduce natural prebiotics, which are “a substrate that is selectively utilized by host microorganisms conferring a health benefit”, such as FOS (fructooligosaccharides), GOS (galactooligosaccharides), and fibers, with possible changes in intestinal microbiota which lack the substrate for fermentation and for its natural metabolic activity [74,75,76,77]. This can decrease the production of SCFAs and their important protective and trophic activity on the colonocytes.

Some studies, by means of different methods, have investigated the effect of a four-week LFD on stool microbiota in patients with IBS.

Three studies showed that an LFD led to a reduction in *Bifidobacteria* concentration. Staudacher et al. studied 41 IBS patients, of which 19 were treated with the LFD and 22 continued their habitual diet [27]. A stool sample was collected at baseline and after four weeks of treatment for analysis of gut microbiota and SCFA dosage. Using fluorescence in situ hybridization (FISH), it was shown that there were no differences in total bacteria, *Bacteroides-Prevotella, E. rectale-C. coccoides, F. prausnitzii,* or *Lactobacillus-Enterococcus* concentrations at baseline or after the LFD. In addition, no difference in fecal SCFA levels was found in the two groups at baseline or after the LFD. On the contrary, a six-fold reduction in the concentration of luminal *Bifidobacteria* was found after the four-week LFD.

Halmos et al. investigated the effect of an LFD in quiescent Crohn’s disease and IBS [56]. They observed that the absolute and relative bacterial abundance of *Akkermansia muciniphila* and *Clostridium cluster XIVa* were reduced with the LFD compared to a typical Australian diet. Moreover, they did not discover any significant differences in absolute and relative abundance of *Lactobacilli* and *Bifidobacteria spp*. between the two controlled diets.

In another study, Halmos et al. analyzed 27 patients with IBS and six healthy subjects treated with an LFD or a typical Australian diet [78]. They found the same results regarding the absolute and relative bacterial abundance, with a reduction in *Clostridium Cluster IV* and *F. prausnitzii* compared to controls, the latter being a butyrate-producing bacterium. The reduction of *F. prausnitzii* has been found in many studies on IBD patients, and its decrease could potentially be harmful to the integrity of the intestinal mucous barrier [79].

Chumpitazi et al. studied a small number of children with IBS [80]. They found that neither the richness of the stool bacterial community, defined as the number of unique operational taxonomic units (OTUs), nor the related α-diversity metrics differed between the baseline and during the LFD periods. However, they observed an increased abundance of *Clostridiales* and a decreased abundance of *Bacteroidetes* during the LFD period.

In another trial, Chumpitazi et al. studied the stool microbiome in IBS children using metagenomic sequencing methods [31]. Responders to the LFD had a different microbiome composition at baseline, which was characterized by increased abundance of saccharolytic capacity species. Non-responders were enriched at baseline only in the genus of *Turicibacter* from the family *Turicibacteraceae*.

Mcintosh et al. performed a controlled, single-blind study on 40 IBS patients (20 treated with LFD and 20 with an HFD) for three weeks [18]. They used metagenomic sequencing methods to assess stool microbiota composition and, unlike the studies that showed a reduction in *Bifidobacteria* after an LFD, they found that the LFD increased *Actinobacteria* richness and diversity compared to the HFD.

Finally, Harvie et al. examined gut microbiota after the reintroduction period [43]. Similarly to Chumpitazi, McIntosh, and Halmos, they found that the LFD did not reduce microbiota diversity.

In conclusion, studies investigating gut microbiota and its metabolic products show mixed, and sometimes conflicting, results. This is probably due to the different study protocols and to the heterogeneity of the FODMAP content in the LFD suggested in the different countries and in the comparator diets. It could also be due to the different sample collection, storage, and analysis methodologies. Moreover, the simple analysis of feces cannot reliably reveal the real GI tract picture. Other approaches, such as metagenomics, transcriptomics, and proteomics, could provide more reliable information [43].

### 3.4. Constipation

Reduction in the FODMAP content may also result in a simultaneous reduction in fiber intake if wholegrain wheat products or high FODMAP fruit and vegetables are not replaced with suitable low-FODMAP alternatives. A reduction in FODMAPs could also reduce osmotic fluid transit into the gut lumen, increasing the likelihood of constipation. These effects could be particularly harmful for IBS patients with ongoing constipation. The intervention of a skilled nutritionist is essential to suggest possible solutions, as long as they are compatible with the LFD.

Some studies have shown that during the LFD, there was a decreased intake of dietary fiber [30,43], whereas other studies have reported an intake of fiber that was not dissimilar to the usual diet [27,32,81].

Particularly, in the study by Bellini et al., IBS patients followed by a skilled nutritionist did not decrease the daily fiber intake using lightly fermentable high-fiber alternatives such as oat and rice bran [66]. The patients improved their fecal consistency with an increased number of them reporting 3-4-5 types of feces, according to the Bristol stool chart, after a two-month LFD. The same results were reported in a study by Maagard et al.: The proportion of IBS patients with normal stools increased by 41% after the LFD [41]. Recently, O’Keeffe also showed that there was no significant difference in fiber content between a habitual diet and an aLFD [42].

In any case, after the strict LFD period, the reintroduction of “non-dangerous” FODMAPs, especially GOS and FOS, increases the fiber intake. Also, patients who had harder feces during a strict LFD reported softer stools, as shown by Staudacher et al. in 67% of constipated IBS patients [37,81].

Indeed, if the LFD is correctly administered, it maintains an adequate amount of fiber, so the risk of constipation is reduced.

### 3.5. Nutritional Adequacy 

One of the main questions that detractors of the LFD pose is its potential nutritional inadequacy because it is a restrictive diet [21,82].

The most frequent issues are:

- The exclusion of carbohydrates rich in fructans may lead to a reduction in carbohydrate, fiber, and iron intake [42];
- The lower amount of kcalories of the LFD may lead to an excessive weight loss;
- The exclusion of several types of vegetables may lead to a reduction in natural antioxidants, such as flavonoids, carotenoids, and vitamin C, or phenolic acid and anthocyanins;
- The exclusion of dairy products may favor calcium deficiency, both because they are the main food source and because lactose acts as a promoter of its absorption.

Bohn et al. performed a multi-center, parallel, randomized, controlled single-blind study, where a dietitian gave verbal instructions to patients regarding dietary advice [30]. They found lower energy and lower carbohydrate and fiber intake in 33 patients treated with the LFD and in 34 treated with a traditional IBS diet (both diets lasting four weeks). There was a greater reduction in the LFD group. Reduced protein and fat intake were only found in patients treated with the LFD.

Harvie et al. found a decrease in energy and fiber intake during the treatment with an LFD [43]. After the reintroduction of FODMAPs up to the patient’s tolerance level, the authors found that both energy and fiber increased to levels similar to those of the habitual diet.

Staudacher showed that four weeks of LFD decreased levels of carbohydrates, starch, total sugars, calcium, and iron intake in 19 IBS patients in comparison to a habitual diet [27]. A possible explanation could be the inadequate inclusion of lactose-free products or calcium–rich alternatives in patients treated with the LFD.

Eswaran et al. compared an LFD (45 patients) to a diet based on NICE guidelines (39 patients) for four weeks [32]. They found a lower intake of carbohydrates and lactose in the group treated with the LFD with respect to the modified NICE group, but no differences in energy, protein, fat, or fiber intake.

In a recent study, the same group found that an LFD, compared to a modified NICE diet, caused a reduction in seric levels of thiamin, riboflavin, calcium, and daily sodium intake [83]. However, after the micronutrient consumption was energy-adjusted, only the riboflavin decreased compared to the mNICE diet. Micronutrient intakes may be quantified as absolute intake and energy-adjusted intake, which is the amount of the micronutrient per 1000 kcals [84]. The number of patients who met their dietary reference intakes did not change, with the exception of thiamin and iron in the LFD arm.

On the contrary, Staudacher et al., comparing dietary intake in patients treated with sham dietary advice and LFD advice, observed no differences in energy, protein, fat, carbohydrate, starch, or sugar intake [34].

Bellini et al. showed that an eight-week LFD, monitored by a skilled nutritionist, improved IBS symptoms with no changes in energy, macronutrients, or fiber intake [66]. No clinically relevant changes were found in blood tests and no effects were detected regarding nutritional status and body composition, which were evaluated for the first time by means of bioelectrical impedance vector analysis (BIVA).

Also, O’Keeffe showed that there were no significant differences regarding energy and nutrient intakes between a habitual diet and an aLFD, with higher levels of folate and vitamin A in the aLFD [42]. Moreover, they observed that total carbohydrate and calcium intakes did not differ between a habitual diet and an aLFD.

Furthermore, there are few studies on LFDs in children and its application remains controversial due to the potential psychological and nutritional risks of a restrictive diet, especially in a developing child. Additional studies are needed before recommending a LFD in children with IBS [85].

In conclusion, because no single food group is completely eliminated during the LFD, as outlined by Dugum and by Staudacher, it is unlikely that patients would encounter a significant and dangerous nutritional imbalance, especially when the diet is monitored by a health professional familiar with this type of diet [72,86].

### 3.6. Precipitating Eating Disorder Behavior 

When prescribing a diet for IBS, it is necessary to remember that a relatively high percentage of patients show psychological comorbidities [87,88,89]. It is therefore not improbable that recommending a restrictive diet will prompt or reinforce an eating disorder. Although Mari et al. reported that these kinds of patients have greater adherence to an LFD, elimination diets should be avoided in patients with eating disorders, such as orthorexia nervosa and/or avoidant/restrictive food intake disorder [90]. These patients have an obsessive focus on food that compromises their social and work life [91].

It is essential to identify patients with the risk of eating disorders using adequate tools such as the SCOFF questionnaire [92].

### 3.7. Long-Term Efficacy

An LFD, given that it is an exclusion diet, should be undertaken for limited periods of time. After the two- to six-week restrictive phase, the patients must be tested to detect which FODMAPs they can safely reintroduce into their diet. This is to avoid possible nutritional imbalance, to improve nutritional adequacy, and to start a more relaxed LFD, eliminating only the real “trigger” foods. Studies on these phases of the LFD are still lacking in the current literature. There are only seven studies investigating the effect of the LFD in terms of long-term efficacy. Five of them [38,40,41,42,43] included a health professional delivering education, whereas two of them did not clarify this [37,39]. There is high variability in study design with respect to the definition of dietary protocols, FODMAP reintroduction, and reliable assessment tools. This limits interstudy comparison and the possibility of generalizing results [92].

A recent retrospective cross-sectional study by Weynants et al. conducted on 90 patients found no significant difference regarding Quality of Life [44]. However, patients who followed an LFD had less severe abdominal pain in the long-term.

In conclusion, studies on long-term efficacy are still lacking and we need to determine LFD therapeutic activity in such a chronic and recurrent disease as IBS.

## 4. Hopes for the Future

Despite the high number of published papers and the amount of data available on LFDs, this kind of diet is still very new, and we have great expectations for its development and improvement. The foreseeable future will probably bring us very important knowledge with respect to different fields, i.e., pathophysiological aspects, clinical indications, and implementation strategies.

It is important that future research helps clinicians better identify patients who can benefit from an LFD and those for whom the diet is unsuitable. In this regard, predictors of response to LFDs remain under investigation, with preliminary data supporting the possible role of fecal microbiota and/or fecal volatile organic compound (VOCs) profiling [92,93].

It is also hoped that there will be more adequate training of health professionals who have to guide patients through the different phases of an LFD. A graded reintroduction of FODMAPs is needed to determine individual tolerance [64]. In addition, to make the LFD more accessible, it is necessary to introduce new methods into clinical practice for patient education beyond the one-to-one interview with a qualified dietician. The more widespread use of dietician-led groups and online apps (see the smartphone application created by Monash University) should play an important role in the foreseeable future [92,94].

Moreover, further studies should clarify whether the restriction phase can be shortened to less than the four to eight week period which we currently use in our practice, given the rapid results in terms of symptom improvement often obtained. This could further minimize the risks of nutritional inadequacy that a restriction diet may have and facilitate patients’ compliance. It is already clear that the length of time of the restrictive phase can be adapted depending on the time required for an adequate symptom response.

It will be necessary to implement studies on the food composition and the measurements of FODMAP content in order to improve the quality of food labeling. This will be very helpful in guiding the consumers/patients in their choice.

It is also mandatory that future studies use common and shared outcome measures in terms of symptom severity scores and quality of life questionnaires, as this will enable a reliable comparison between different studies. It is also to be hoped that in the near future, an easy and reliable tool to assess patients’ adherence will be available, especially to evaluate the patients in the medium- and long-term, i.e., during the aLFD period, after the reintroduction phase [90].

It is of paramount importance that future research focuses on the modality of the reintroduction phase, that is, when to commence the “starting” foods, and how long and to what extent they need to be tested. It is crucial to assist patients in identifying specific dietary triggers, reducing the level of dietary restriction required, and increasing the prebiotic intake [68,95].

Also, the aLFD phase, following the reintroduction phase, deserves further investigation. During this long-term phase, when the patient reaches a certain stability in their personalization of the diet, the analysis of microbiota and metabolome could be very important to assess the safety of the LFD. This may possibly ease the concerns of the LFD being a cause of gut dysbiosis. With regards this aspect, a supplementation with probiotics, as suggested by Staudacher et al., could very well be a promising option to prevent possible changes in microbiota and metabolome [34].

Finally, given the proven efficacy of the LFD diet within the context of functional gastrointestinal symptoms, it is still necessary to broaden the field of study to other diseases that often have some symptoms in common with IBS and which could probably benefit from an LFD. These include IBD, functional esophageal and duodenal disorders, exercise-related gastrointestinal symptoms, radiation-induced enteropathy and nonceliac gluten sensitivity [55,57,96,97,98,99,100].

## 5. Conclusions

An LFD is currently suggested as an effective treatment for IBS despite the lack long-term, randomized controlled studies on large numbers of patients. To date, many existing studies are of a low quality.

Some potential limitations and concerns of LFDs have been raised, such as nutritional adequacy, cost, and difficulty in teaching, learning, and continuing the diet. Most of these problems are amplified in patients who follow the diet without clear professional advice. Therefore, they can be easily resolved with the presence of a skilled nutritionist who is able to explain the different phases of LFD and to ensure nutritional adequacy and the patient’s compliance. Moreover, it would be desirable to improve the food analysis regarding FODMAP content while also considering the varied eating habits of different populations in different countries. This would enable a better labeling of foods, thus improving the patients’ knowledge regarding possible “dangerous foods”. Further studies should focus on new methods of teaching and learning LFDs on its different phases and on predictors of response. Future research will therefore need to address some issues of paramount importance, such as the role of LFD on the gut microbiota and research into diagnostic markers such as VOCs in order to identify which patients could benefit from this diet. Possible new LFD fields of application, other than functional GI disorders, should be investigated in the foreseeable future.

## Figures and Tables

**Table 1:** LFD efficacy in IBS.

	Trial Characteristics	Methods	Length of Follow-Up	Evaluated Parameters	Results	Grade of Evidence
**Short-term efficacy**						
McIntosh et al. [18] 2017	LFD = 19 HFD = 18 Rome III	Single blinded parallel	3 weeks	IBS-SSS	Lower IBS-SSS in LFD group for gastrointestinal symptoms and abdominal pain.	Low
Ong et al. [26] 2010	LFD or HFD IBS = 15 Healthy controls = 15 Rome III	Single blinded, crossover	2 days	Likert scale (GI symptoms severity)	IBS patients under HFD had more severe symptoms compared to those on LFD.	Low
Staudacher et al. [27] 2012	LFD = 19 Habitual diet = 22 Rome III	Single blinded, controlled	4 weeks	GSRS BSC	LFD group had better adequate symptom control, lower stool frequency, less abdominal pain, and less overall symptoms.	Low
Pedersen et al. [28] 2014	LFD = 42 Probiotic = 41 Habitual diet = 40 Rome III	Unblinded parallel	6 weeks	IBS-SSS IBS-QOL	Greater reduction in IBS-SSS in LFD group compared to habitual diet. No differences in IBS QOL.	Very low
Halmos et al. [29] 2014	LFD or Typical (Australian) diet IBS = 30 Healthy controls = 8 Rome III	Single blinded, controlled crossover	21 days	VAS (GI symptoms severity) KSC FWC	Lower VAS in LFD group. Lower stool frequency and lower KSC score in IBS-D during LFD.	Low
Bohn et al. [30] 2015	LFD = 33 NICE = 34 Rome III	Single blinded, multicentre parallel, controlled	4 weeks	IBS-SSS HADS BSC Visceral sensitivity index	IBS symptoms reduced in both diets, with no difference between groups.	Low
Chumpitazi et al. [31] 2015	Pediatric patients LFD = 16 TACD = 17 Rome III	Double blinded, crossover	48 hours	Pain and stool diary Likert scale (Pain severity and associated GI symptoms) BSC	Fewer abdominal pain episodes and less severity during LFD. Total composite GI score lower in LFD.	High
Eswaran et al. [32] 2016	LFD = 45 mNICE = 39 Rome III	Unblinded parallel	4 weeks	AR BSC	Greater reduction in abdominal pain and stool consistency in LFD group. No differences between groups regarding adequate symptom relief.	Very low
Laatikainen et al. [33] 2016	Rye bread = 43 Low FODMAP rye bread = 44 Rome III	Double blinded controlled crossover	4 weeks	IBS-SSS VAS (GI symptoms severity) IBS-QOL	Less abdominal pain, flatulence, stomach rumbling, and intestinal cramps in the Low-FODMAP rye bread group.	High
Staudacher et al. [34] 2017	Sham diet/placebo = 27 Sham diet/probiotic = 26 LFD/placebo = 24 LFD/probiotic = 27 Rome III	Single blinded, multicentre, placebo-controlled,	4 weeks	GSRS IBS-SSS BSC IBS-QOL SF-36	Lower IBS-SSS and better IBS QOL in LFD group.	High
Hustoft et al. [35] 2017	LFD and maltodextrin = 20 LFD and FOS = 20 Rome III	Double blinded, placebo-controlled, crossover	9 weeks	IBS-SSS VAS (associated symptoms) AR	Lower IBS-SSS and more patients reporting symptom relief in the group supplemented with maltodextrin	High
Peters et al. [36] 2015	LFD = 24 Hypnotherapy = 25 Combined = 25 Rome III	Unblinded	6 weeks	VAS (GI symptoms severity) IBS-QOL HADS STPI	Lower VAS in LFD and hypnotherapy. IBS-QOL improved in all groups with no statistical differences.	Very low
**Long-term efficacy**						
Staudacher et al. [37] 2011	LFD = 43 NICE diet = 39 No aLFD Dietitian-led education	Retrospective observational	2–6 months	Likert scale (symptom changes and satisfaction with dietary advice)	LFD group reported improvement in bloating, abdominal pain, flatulence, nausea, and energy levels, and more satisfaction with the treatment.	Very low
Peters et al. [38] 2016	LFD + aLFD = 24 Hypnotherapy = 25 Combination = 25 Dietitian-led education	Unblinded, randomized	6 weeks + 6 months	VAS IBS-SSS STPI HADS IBS-QOL	Improvements in overall symptoms for hypnotherapy, LFD and combination, maintained at 6 months. Hypnotherapy superior regarding psychological indices.	Very low
Schumann et al. [39] 2018	LFD for 12 weeks + aLFD = 29 Yoga 12 weeks = 30 Dietitian-led education	Single blinded randomized controlled trial	6 months	IBS-SSS IBS-QOL SF-36 HADS CPSS PSQ BAQ BRS AR	IBS-SSS scores decreased both for LFD and yoga, with no statistically significant group differences. HADS scores were lower in yoga group, especially on the subscale for anxiety.	Low
de Roest et al. [40] 2013	LFD = 90 Dietitian-led education	Prospective observational	15.7 (±9.0) months	GI symptom rating scale Likert scale (symptoms intensity and adherence)	Positive change in most of the investigated symptoms, including abdominal pain, bloating, flatulence, and diarrhea. Fructose malabsorption was associated with response to the diet. 75.6% were adherent to LFD.	Very low
Maagaard et al. [41] 2016	IBS = 131 IBD = 49 LFD for 6-8 weeks + aLFD = 180 Dietitian-led education	Retrospective cross-sectional	16 months (range: 2–80)	VAS FARS BSC IBS-SSS IBS-QOL SIBDQ	Partial or full efficacy of bloating and abdominal pain. One third were adherent to the diet. LFD was reported to be more expensive and complicated than usual diet.	Very low
O’Keeffe et al. [42] 2018	NICE IBS criteria LFD for 6 weeks + aLFD = 103 Dietitian-led education	Prospective observational	6–18 months	Global symptom response GSRS BSC Likert scale (acceptability and impact on daily life)	Abdominal pain, bloating and flatulence decreased at long-term follow up. Satisfactory symptom relief was reported at follow-up. aLFD was found to be more expensive and difficult than habitual diet.	Very low
Harvie et al. [43] 2017	LFD = 23 Habitual diet = 27 aLFD = 23 LFD = 27 Dietitian-led education	Randomized, parallel, cross-over	6 months	IBS-SSS IBS-QOL	Lower reduction of IBS-SSS and better QoL in LFD (3 months) and sustained by aLFD (6 months).	Low
Weynants et al. [44] 2019	LFD for 6–8 weeks + aLFD = 90 Dietitian-led education	Retrospective cross-sectional	49–168 weeks	IBS-QOL IBS-SSS Self-developed adherence and symptoms questionnaire	Patients who still followed the diet had less severe abdominal pain. 80% of patients were adherent to the LFD. No significant difference in QOL was found.	Very low

aLFD: Adapted LFD; AR: Adequate symptom Relief; BAQ: Body Awareness Questionnaire; BRS: Body Responsiveness Questionnaire; BSC: Bristol Stool Chart; CPSS: Cohen Perceived Stress Scale; FARS: FODMAP Adherence Report Scale; FODMAPs: Fermentable Oligo-, Di- and Monosaccharides and Polyols; FOS: Fructooligosaccharides; FWC: Fecal Water Content; GI: Gastrointestinal; GSRS: GI Symptoms Rating Scale; HADS: Hospital Anxiety and Depression Scale; HFD: High FODMAP Diet; IBD: Inflammatory Bowel Disease; IBS: Irritable Bowel Syndrome; IBS-D: Diarrhea predominant Irritable Bowel Syndrome; IBS-QOL: Irritable Bowel Syndrome Quality of Life; IBS-SSS: Irritable Bowel Syndrome Severity Scoring System; KSC: King’s Stool Chart; LFD: Low-FODMAP Diet; LGG: Lactobacillus rhamnosus GG; mNICE: Modified National Institute for Health and Clinical Excellence; NICE: National Institute for Health and Clinical Excellence; n.a.: not assessed; PSQ: Perceived Stress Questionnaire; SF-36: Short Form Health Survey; SIBDQ: Short IBD Questionnaire; STPI: State-Trait Personality Inventory; TACD: Typical American Childhood Diet; VAS: Visual Analogue Scale.
